# Phenotypic Characterization and Marker–Trait Association Analysis Using SCoT Markers in Chrysanthemum (*Chrysanthemum morifolium* Ramat.) Germplasm

**DOI:** 10.3390/genes16060664

**Published:** 2025-05-29

**Authors:** Fenglan Wang, Xiuzhe Chen, Zifeng Huang, Lisha Wei, Jun Wang, Shuang Wen, Yang Liu, Yiwei Zhou

**Affiliations:** 1 College of Horticulture and Landscape Architecture, Zhongkai University of Agriculture and Engineering, Guangzhou 510225, China; 2 Zhonghua Modern Agriculture Research Institute, Huadu District, Guangzhou 510800, China; 3 Dongguan Research Center of Agricultural Sciences, Dongguan 523086, China; 4 College of Architectural Engineering, Shenzhen Polytechnic University, Shenzhen 518055, China; 5 Guangdong Provincial Key Laboratory of Ornamental Plant Germplasm Innovation and Utilization, Environmental Horticulture Research Institute, Guangdong Academy of Agricultural Sciences, Guangzhou 510640, China

**Keywords:** chrysanthemum, phenotypic trait, genetic diversity, SCoT, association analysis

## Abstract

**Background:** Chrysanthemum is an economically important ornamental species whose genetic diversity assessment forms the foundation for effective breeding programs. Methods: Phenotypic characterization of 12 traits (7 quantitative and 5 qualitative traits) was conducted alongside SCoT marker analysis to assess genetic diversity and perform marker–trait association analysis in 65 chrysanthemum accessions. Results: Quantitative traits showed 14.81–26.43% variation (peduncle length most variable), while qualitative traits exhibited Shannon–Weiner indices of 0.23–2.28 (flower color most diverse). Phenotypic analyses consistently grouped accessions into two clusters. SCoT markers generated 160 bands (159 polymorphic; 6.957 bands/marker) with high PIC values (0.408–0.896). Molecular analyses also revealed two genetic groups, though with partial discordance to phenotypic clusters. Eight significant marker–trait associations were identified, linking SCoT28/3/30/31/35/20/14/36 to flowering duration, plant height, peduncle diameter, flower color, and pest resistance traits. Conclusions: The study revealed substantial diversity in local chrysanthemum germplasm, with SCoT markers effectively capturing genetic variation. While phenotypic and molecular groupings showed partial mismatch, identified marker–trait associations (e.g., SCoT28 linked to flowering duration) provide practical tools for marker-assisted breeding.

## 1. Introduction

Chrysanthemum (*Chrysanthemum morifolium* Ramat.), a perennial herbaceous species in the Asteraceae family, holds dual significance as one of China’s ten most celebrated traditional flowers and one of the world’s four major cut flowers, prized for its exceptional ornamental value [1,2]. In global floriculture trade, it ranks as the second most economically important cut flower after roses [3,4].

Comprehensive evaluation of phenotypic variation facilitates parental selection in breeding programs while elucidating evolutionary adaptation mechanisms. Deciphering the genetic architecture underlying phenotypic variation enables identification of superior alleles and informs molecular breeding strategies [5]. The species exhibits remarkable phenotypic plasticity attributable to its extensive genetic diversity [5]. Previous investigations have documented various aspects of chrysanthemum diversity. Sun et al. [6] established the significant impact of leaf biomass on cut-flower quality, providing a theoretical framework for optimizing leaf–stem ratios in breeding. Miler et al. [7] reported phenotypic instability in vegetative and reproductive traits among in vitro-regenerated plants, while Lu et al. [8] systematically categorized floral pigmentation patterns across 273 pot cultivars into 6 distinct groups. Given the vast repository and environmental sensitivity of trait expression, continuous diversity assessment of regional varieties remains imperative for developing locally adapted varieties.

Start-codon-targeted (SCoT) polymorphism, developed by Collard et al. [9], is a PCR-based molecular marker technique that utilizes 18–22 bp primers designed from conserved regions flanking the translation initiation codon (ATG). Targeting these functionally significant regions involved in gene expression regulation, SCoT markers demonstrate superior potential for functional marker development and gene characterization [10,11,12,13]. Compared to conventional markers (e.g., ISSR and RAPD), SCoT offers distinct advantages, including technical simplicity, reproducibility, cost-effectiveness, and stronger associations with functional genes, making it particularly suitable for genetic diversity studies, cultivar identification, phylogenetic analysis, and gene mapping [14]. While next-generation sequencing (NGS) methods (e.g., GBS, RAD-seq, and SLAF-seq) provide greater throughput and genome-wide coverage for high-density SNP detection [15,16,17], their higher costs render SCoT a preferred choice for resource-limited studies or species with insufficient genomic resources.

SCoT markers have demonstrated significant utility in chrysanthemum genetic studies. Li et al. [18] reported superior gene diversity and polymorphism information content (PIC) values with SCoT markers compared to SRAP and EST-SSR markers when analyzing 159 cut-flower cultivars. Feng et al. [19] reported high PIC values using 32 SCoT markers across *C. morifolium* accessions. Samarina et al. [20] successfully discriminated 95 core chrysanthemum collections based on morphological traits using 36 SCoT markers. Additionally, SCoT has proven valuable for assessing genetic stability in chrysanthemum materials [21,22], collectively establishing its efficacy for genetic variation analysis. To our knowledge, systematic phenotype–trait association analysis in chrysanthemum using SCoT markers remains largely unexplored.

To systematically evaluate the phenotypic and genetic diversity of local chrysanthemum germplasm in Guangzhou and establish a foundation for parental selection and marker-assisted breeding, this study combines phenotypic characterization with SCoT marker analysis across 65 representative accessions. The integrated approach identifies genetic loci associated with key ornamental traits, providing essential insights for future genetic enhancement programs.

## 2. Materials and Methods

### 2.1. Plant Materials

All chrysanthemum germplasm used in this study was obtained from the Chrysanthemum Germplasm Resource Nursery (113°32′ E, 23°47′ N) in Huadu District, Guangzhou. Sixty-five morphologically distinct cultivars were selected and cultivated under protected greenhouse conditions at the experimental site in August 2023, with environmental parameters maintained at a 25–30 °C average temperature, 70–85% relative humidity, and 12–14 h natural photoperiod. Apical shoot cuttings (approximately 5 cm in length) were collected from healthy mother plants and transplanted into 110 mm plastic pots containing a peat:perlite (3:1 *v*/*v*) growing medium. Six biological replicates were maintained per cultivar. Phenotypic characterization was performed at the full flowering developmental stage. Detailed information for all samples is provided in Table S1.

### 2.2. Phenotypic Trait Measurement and Analysis

Following China’s agricultural industry standard, *Guidelines for the conduct of tests for distinctness, uniformity, and stability—Chrysanthemum* (NY/T 2228-2012) [23], seven quantitative traits (plant height, peduncle length, peduncle diameter, leaf length, leaf width, flower diameter, and flowering duration) and five qualitative traits (peduncle color, flower color, petal type, aphid resistance, and leaf-miner resistance) were evaluated. Morphological measurements were conducted during peak flowering using vernier calipers, rulers, and tape measures, with a minimum of five replicates per trait. Detailed measurement protocols for the 12 traits are provided in Table S2. Basic descriptive statistics, Pearson correlation analysis, hierarchical cluster analysis (HCA), and principal component analysis (PCA) were performed using built-in functions in R 4.3.1 [24]. The genetic diversity of traits across groups was assessed using the Shannon–Wiener index (*H*), computed as Equation (1):

(1) $$H=-\sum_{i=1}^{S}PilnPi$$

In Equation (1), *S* represents the total number of chrysanthemum accessions, and *Pi* denotes the relative frequency of the *i*-th phenotypic variant within the sample population.

### 2.3. DNA Extraction

Genomic DNA was extracted from young leaves using the FastPure Plant DNA Isolation Mini Kit (Vazyme Biotech, Nanjing, China) according to the manufacturer’s protocol. DNA concentration and purity were determined spectrophotometrically using a Nanodrop ND-1000 (Thermo Fisher Scientific, Waltham, MA, USA). All DNA samples were normalized to a working concentration of 30 ng/μL using 1× TE buffer (10 mM Tris-HCl, 1 mM EDTA, pH 8.0) and stored at −20 °C until further analysis.

### 2.4. SCoT-PCR Amplification

Thirty-six SCoT primers originally designed by Collard and Mackill [9] were initially screened, of which twenty-three produced reproducible polymorphic amplification patterns. PCR amplifications were performed in 20 μL reaction volumes containing 10 μL 2× *Taq* Master Mix (including DNA polymerase, dNTPs, and reaction buffer), 1 μL primer (10 μM), 1 μL of template DNA (30 ng), and 8 μL of sterile distilled water. The thermal cycling protocol consisted of initial denaturation at 94 °C for 3 min, followed by 33 cycles of denaturation at 94 °C for 10 s, primer-specific annealing at 50–65 °C for 20 s (optimized for each primer pair), and extension at 72 °C for 10 s, with a final extension at 72 °C for 5 min. Amplification products were separated by electrophoresis through 1% (*w*/*v*) agarose gels at 110 V for 45 min, stained with ethidium bromide, and visualized under UV illumination. Polymorphic bands were scored as binary data (1 = present and 0 = absent).

### 2.5. Genetic Diversity and Population Structure Analysis

Polymorphism information content (PIC) values were calculated, as described by Nagy et al. [25]. Pairwise genetic distances were computed using PowerMarker software v3.25 [26], and a neighbor-joining (NJ) phylogenetic tree was constructed and visualized in MEGA 7.0 [27]. Population structure was inferred through Bayesian clustering analysis implemented in STRUCTURE v2.3.4 [28], with the number of presumed subpopulations (*K*) ranging from 1 to 10. For each *K*-value, 10 independent runs were performed using 100,000 Markov chain Monte Carlo (MCMC) iterations after a burn-in period of 50,000 iterations. The optimal *K*-value was determined by evaluating ΔK statistics using structureHarvester v0.6.93 [29]. Resulting Q-matrices from replicate runs were aligned and merged with CLUMPP v1.1.2 [30]. Principal coordinate analysis (PCoA) was conducted using GenAlEx 6.5 [31].

### 2.6. Association Analysis

Marker–trait associations were identified in TASSEL 5.0 [32] with a mixed linear model (MLM) that incorporated both population structure (Q-matrix) and kinship relationships as covariates to minimize false positives. Statistical significance of associations between 12 phenotypic traits and 23 SCoT markers was determined at a threshold of *p* < 0.01 after multiple testing correction.

## 3. Results

### 3.1. Phenotypic Diversity Analysis

#### 3.1.1. Variation in Quantitative Traits

**Figure 1:**
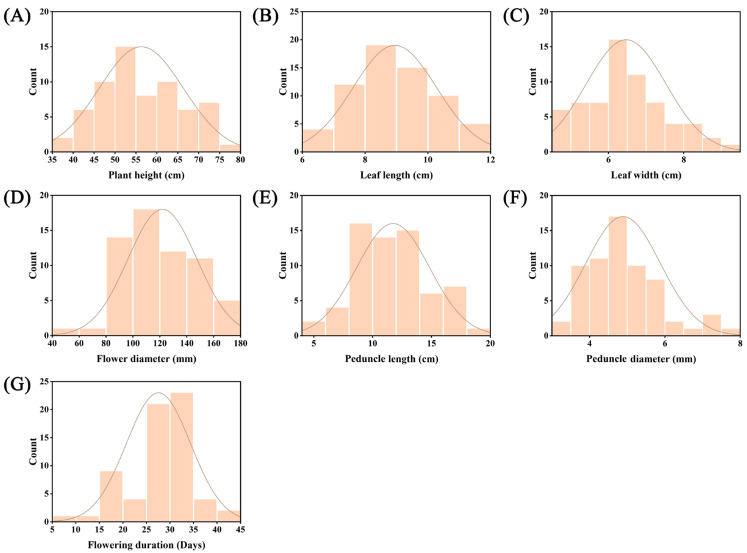
Frequency distribution histograms of seven quantitative traits among 65 chrysanthemum accessions: (**A**) plant height, (**B**) leaf length, (**C**) leaf width, (**D**) flower diameter, (**E**) peduncle length, (**F**) peduncle diameter, and (**G**) flowering duration.

Morphological variation was evaluated among 65 chrysanthemum accessions by analyzing 7 quantitative traits, including their minimum, maximum, mean, standard deviation, and coefficient of variation (CV). Frequency distributions of all seven quantitative traits exhibited approximately normal distributions (Figure 1). The CV values ranged from 14.81% to 26.43%, reflecting substantial variation among cultivars (Table S3). Peduncle length (mean = 11.74 cm; range = 5.75–19.26 cm; CV = 26.43%) exhibited the highest variability, whereas leaf length (CV = 14.81%) displayed the greatest stability. Intermediate variation was observed in flowering duration (8–44 days; CV = 24.76%), flower diameter (57.37–175.83 mm; CV = 19.79%), and peduncle diameter (3.18–7.87 mm; CV = 20.30%). In contrast, plant height (CV = 17.86%) and leaf width (CV = 16.35%) demonstrated relatively lower variation. Notably, the two foliar traits (leaf length and width) consistently showed lower CV values compared to other traits, suggesting greater genetic stability in leaf morphology across cultivars.

#### 3.1.2. Diversity of Qualitative Traits

**Figure 2:**
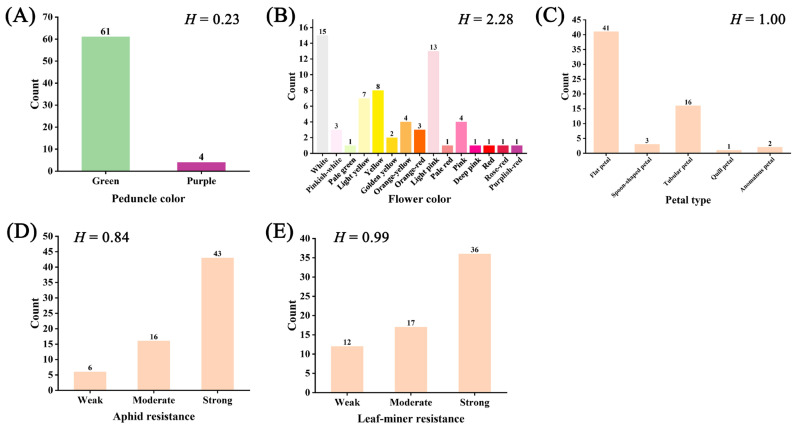
Frequency distribution of 5 qualitative traits among 65 chrysanthemum accessions: (**A**) peduncle color, (**B**) flower color, (**C**) petal type, (**D**) aphid resistance, and (**E**) leaf-miner resistance. *H*: Shannon–Wiener index.

The distribution of 5 qualitative traits was analyzed across 65 chrysanthemum accessions. Peduncle color was classified into two primary categories: green (98.5%, predominant) and purple (1.5%). For flower color traits, the 65 accessions exhibited 15 distinct color variations, including white, pinkish-white, pale green, light yellow, yellow, golden yellow, orange-yellow, orange-red, light pink, pale red, pink, deep pink, red, rose-red, and purplish-red. Notably, green varieties were exceptionally rare (1.54%), while white (23.08%) and light pink (20.00%) constituted the most prevalent color phenotypes. Regarding petal type, the observed types included flat petal, spoon-shaped petal, tubular petal, quill petal, and anomalous petal. Among these, flat petals were the most prevalent (63.08%), followed by tubular petals (24.62%). Pest resistance assessments indicated high aphid resistance in 66.15% of accessions and elevated leaf-miner resistance in 55.38%, with intermediate resistance levels observed in 24.61% (aphids) and 26.15% (leaf-miners) of specimens. A significant proportion (18.46%) exhibited low leaf-miner resistance. Shannon–Weiner diversity indices (*H*) for the five traits ranged from 0.23 to 2.28, with flower color demonstrating the highest diversity (*H* = 2.28) and peduncle color the lowest (*H* = 0.23; Figure 2). Only flower color and petal type exhibited substantial diversity (*H* ≥ 1), while the remaining traits showed limited variation.

#### 3.1.3. Multivariate Analysis

**Figure 3:**
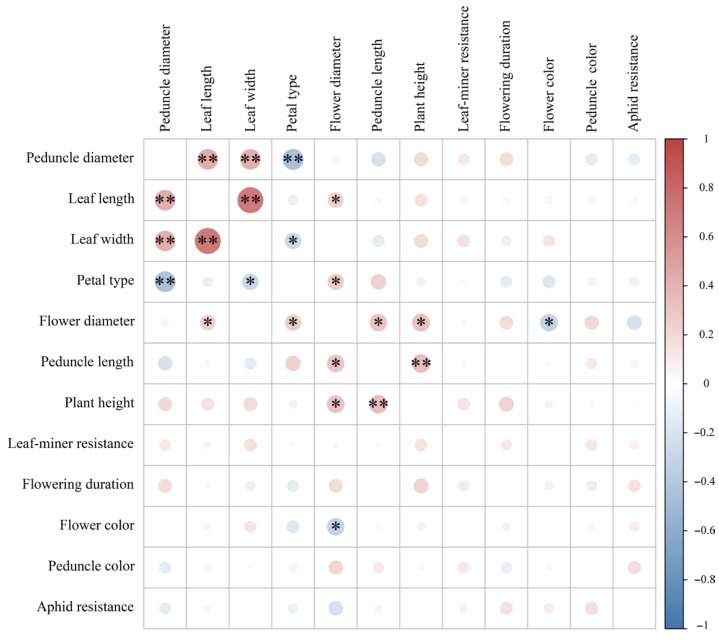
Pearson correlation matrix among 12 phenotypic traits. Asterisks indicate significant correlations (* *p* < 0.05 and ** *p* < 0.01).

Correlation analysis revealed significant associations among the 12 evaluated traits, with Pearson correlation coefficients ranging from −0.446 to 0.435 (Table S4). Notably, peduncle diameter, leaf length, and leaf width exhibited strong positive correlations (*p* < 0.01), while peduncle diameter showed a significant negative correlation with petal type (Figure 3). Flower diameter was positively correlated with both peduncle length and plant height (*p* < 0.05), as well as with leaf length and petal type, but demonstrated an inverse relationship with flower color (Figure 3).

**Figure 4:**
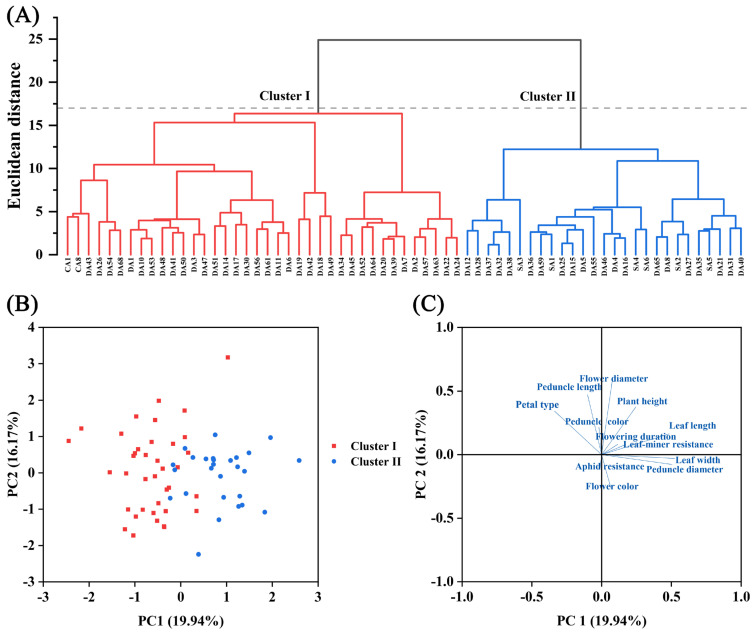
Multivariate analysis of 65 chrysanthemum accessions based on 12 phenotypic traits. (**A**) HCA dendrogram. The gray dashed line indicates the position where the Euclidean distance equals 17. (**B**) PCA score plot. (**C**) PCA loading plot.

Hierarchical cluster analysis (HCA) using a Euclidean distance threshold of 16 delineated the 65 accessions into 2 discrete clusters based on phenotypic traits (Figure 4A). Cluster I (*n* = 38) comprised accessions characterized by reduced plant height, narrower peduncle diameter, smaller leaf dimensions (length and width), abbreviated flowering duration, predominantly purple peduncles, and lighter flower color. Conversely, Cluster II (*n* = 27) contained accessions with greater plant height, larger peduncle diameter and leaf dimensions, prolonged flowering duration, exclusively green peduncles, and darker flower color.

Principal component analysis extracted five principal components with eigenvalues exceeding 1, collectively explaining 67.63% of the total variance (PC1: 19.94%, PC2: 16.17%, PC3: 11.74%, PC4: 10.97%, and PC5: 8.81%; Table S5). The PCA score plot corroborated the HCA clustering, with clear separation of Cluster I and Cluster II accessions along PC1 (Figure 4B). Component loadings analysis revealed that PC1 was strongly influenced by peduncle diameter (loading = 0.51), leaf length (0.48), and leaf width (0.52), while PC2 primarily reflected variation in flower diameter (0.59), peduncle length (0.47), plant height (0.37), and petal type (0.34; Figure 4C and Table S5). These multivariate patterns substantiated the trait correlations identified in prior analyses.

### 3.2. Genetic Diversity Analysis Based on SCoT Markers

#### 3.2.1. Genetic Diversity Parameters

Amplification of 65 chrysanthemum accessions using 23 SCoT primers generated 160 distinct bands, with an average of 6.957 bands per primer (Table 1). Remarkably, 159 bands (99.46%) were polymorphic, with only primer, SCoT24, producing a single monomorphic band. The PIC values ranged from 0.408 to 0.896 (mean = 0.737), demonstrating high levels of polymorphism and substantial genetic diversity within the germplasm. Among these, primer SCoT14 exhibited the highest PIC value, while primers SCoT14, SCoT23, and SCoT35 each produced the maximum number of polymorphic bands (12 bands per primer).

#### 3.2.2. Population Structure, PCoA, and Cluster Analysis

**Figure 5:**
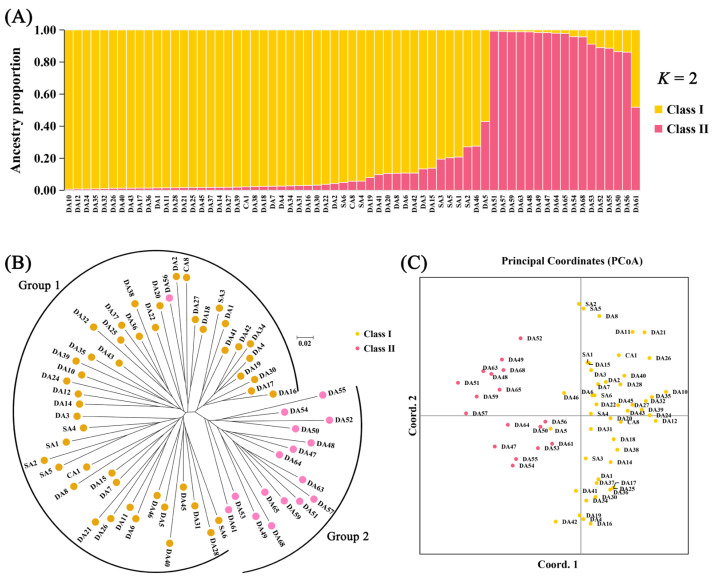
Population genetic analysis of 65 chrysanthemum accessions using 23 SCoT markers. (**A**) Population structure analysis showing ancestry proportions (*K* = 2). (**B**) Neighbor-joining phylogenetic tree. (**C**) PCoA score plot in two dimensions.

Population structure analysis identified optimal clustering at *K* = 2 (determined by maximum delta *K*; Figure S1), categorizing the accessions into two primary groups: Class I (*n* = 48) and Class II (*n* = 17; Figure 5A). NJ cluster analysis yielded comparable grouping patterns, with Group 1 (*n* = 49) comprising 48 accessions from Class I and 1 from Class II, and Group 2 (*n* = 16) exclusively contained Class II accessions (Figure 5B). PCoA results further corroborated this division, revealing distinct separation between Class I and Class II accessions (Figure 5C). Notably, 88.89% of phenotypic Cluster II accessions were classified within genetic Class I, whereas phenotypic Cluster I exhibited mixed ancestry (36.84% from Class II and 63.16% from Class I).

### 3.3. Marker–Trait Association Analysis

**Figure 6:**
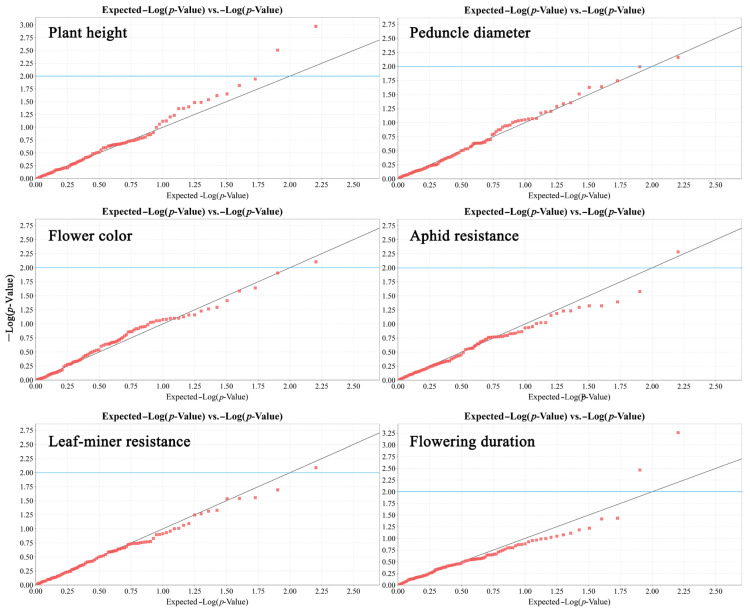
Quantile–quantile (Q–Q) plots of marker–trait associations for six phenotypic traits analyzed using the mixed linear model (MLM) with SCoT markers. Red dots represent individual SCoT markers. Blue line indicates the significance threshold (*p* = 0.01). Markers above the blue line are statistically significant (*p* < 0.01).

Association analysis identified eight significant marker–trait associations (*p* < 0.01) involving six traits, explaining 11.18–20.92% of the observed phenotypic variation (Table 2 and Figure 6). Plant height was associated with SCoT3-band3 (18.53%) and SCoT30-band8 (14.84%). Flowering duration exhibited correlations with SCoT28-band1 (20.92%) and SCoT31-band1 (14.58%). Additionally, significant associations were identified for aphid resistance (SCoT35-band10, 13.32%), peduncle diameter (SCoT20-band1, 12.31%), flower color (SCoT14-band10, 11.81%), and leaf-miner resistance (SCoT36-band1, 11.18%).

## 4. Discussion

Phenotypic diversity constitutes the fundamental basis for genetic improvement in chrysanthemum breeding programs [2]. Although previous investigations have characterized various ornamental and resistance traits across chrysanthemum cultivars [33,34,35], substantial phenotypic variation persists among regional accessions. Our evaluation of 12 ornamental traits across 65 accessions from Guangzhou’s germplasm collection revealed quantitative trait variation coefficients ranging from 14.81% to 26.43% (maximum in peduncle length) and qualitative trait Shannon–Weiner indices spanning 0.23–2.28 (flower color exhibiting maximal diversity). Notably, peduncle length, flowering duration, flower diameter, and color exhibited notable diversity, aiding local cultivar development. Multivariate analysis grouped accessions into two distinct clusters. Similar grouping patterns have been reported in studies investigating inheritance patterns in hybrid populations [36,37], suggesting that strategic crosses between cluster groups could facilitate trait introgression. Nevertheless, the moderate sample size (*n* = 65) may limit broader inferences, necessitating expanded germplasm evaluations to validate marker–trait associations across diverse environments and account for genotype-by-environment (G × E) interactions.

Compared to alternative molecular markers, SCoT technology offers unique advantages independent from prior genomic information, high polymorphism/reproducibility, functional gene proximity, and cost efficiency [9]. From an initial screening of 36 primers, we selected 23 SCoT markers demonstrating exceptional polymorphism (mean 99.46%) with PIC values of 0.408–0.896 (mean = 0.737). Remarkably, 95.7% of markers (22/23) exceeded the 0.5 PIC threshold for high polymorphism [38], indicating greater genetic diversity than reported in comparable studies (PIC 0.37–0.42) [20]. Population structure, NJ clustering, and PCoA consistently segregated accessions into two genetic clusters, though partial discordance with phenotypic groupings underscores the necessity of integrated analysis in parent selection. A key limitation remains the absence of marker validation through segregation analysis or functional studies—a critical next step requiring linkage mapping or transgenic validation.

Association analysis has become instrumental for linking molecular markers to agronomic traits, enabling marker-assisted breeding. In chrysanthemum, researchers have successfully mapped loci governing aphid resistance [35], abiotic stress tolerance [39,40,41], flower color [42], flowering time [37,43], and phytochemical composition [44], using SNP, SSR, and SRAP markers. Fewer studies have leveraged SCoT markers for such analyses. While Samarina et al. [20] noted correlations between SCoT-based clusters and morphological traits, formal association studies were lacking. Our mixed linear model analysis identified eight SCoT markers significantly associated (*p* < 0.01) with six key traits, including SCoT28’s significant association with flowering duration (20.92% variance explained)—potentially implicating photoperiod response genes, consistent with prior chrysanthemum flowering time studies [37,43].

The phenotypic and molecular data generated in this study provide a foundation for optimizing parental selection and breeding strategies in chrysanthemum. To advance genetic improvement of ornamental traits, we propose expanding both natural variation and hybrid populations while incorporating next-generation sequencing (NGS) to develop high-resolution SNP and InDel markers. This approach will facilitate precise gene mapping and marker-assisted selection. Building upon the findings of this study, we propose the following future research directions: (1) validating the identified SCoT markers in biparental populations to confirm trait associations, (2) integrating high-throughput genotyping techniques (e.g., GBS or WGS) to improve marker resolution, and (3) assessing trait stability across diverse environments to account for G × E interactions. These steps will enhance the translation of genetic diversity insights into practical cultivar development.

## 5. Conclusions

This study characterized the phenotypic and genetic diversity of 65 chrysanthemum accessions from Guangzhou, identifying valuable marker–trait associations. Phenotypic evaluation showed significant variation, with peduncle length (CV: 14.81–26.43%) and flower color (Shannon–Weiner index: 0.23–2.28) displaying the highest diversity. Cluster analyses consistently grouped accessions into two distinct phenotypic clusters. Genetic analysis using 23 SCoT markers revealed high polymorphism (99.46%, mean PIC = 0.737), confirming their utility for chrysanthemum genotyping. While population structure analyses similarly identified two genetic groups, their partial mismatch with phenotypic clusters emphasizes the need for combined data interpretation. Eight SCoT markers were significantly associated with six important traits (including flowering duration, plant architecture, flower characteristics, and pest resistance), explaining 11.18–20.92% of the phenotypic variation. These findings provide practical molecular tools for marker-assisted breeding in chrysanthemum improvement programs.

## Figures and Tables

**Table 1:** PCR amplification and polymorphism statistics of 23 SCoT markers across 65 chrysanthemum accessions.

Marker	Sequence (5′-3′)	OAT^1^ °C	Amplified Bands	Polymorphic Bands	Polymorphism Ratio	PIC^2^
SCoT1	CAACAATGGCTACCACCA	55.4	5	5	100.00%	0.769
SCoT2	CAACAATGGCTACCACCC	53.4	6	6	100.00%	0.767
SCoT3	CAACAATGGCTACCACCG	54.1	3	3	100.00%	0.408
SCoT4	CAACAATGGCTACCACCT	52.1	4	4	100.00%	0.708
SCoT7	CAACAATGGCTACCACGG	54.1	5	5	100.00%	0.539
SCoT8	CAACAATGGCTACCACGT	52.6	5	5	100.00%	0.714
SCoT9	CAACAATGGCTACCAGCA	52.7	5	5	100.00%	0.724
SCoT11	AAGCAATGGCTACCACCA	58.1	9	9	100.00%	0.774
SCoT14	ACGACATGGCGACCACGC	66.3	12	12	100.00%	0.896
SCoT16	ACCATGGCTACCACCGAC	63	5	5	100.00%	0.573
SCoT18	ACCATGGCTACCACCGCC	66.5	9	9	100.00%	0.721
SCoT20	ACCATGGCTACCACCGCG	61.8	7	7	100.00%	0.829
SCoT21	ACGACATGGCGACCCACA	69.3	8	8	100.00%	0.822
SCoT22	AACCATGGCTACCACCAC	59.2	5	5	100.00%	0.772
SCoT23	CACCATGGCTACCACCAG	61	12	12	100.00%	0.868
SCoT24	CACCATGGCTACCACCAT	61	8	7	87.50%	0.855
SCoT27	ACCATGGCTACCACCGTG	62	9	9	100.00%	0.879
SCoT28	CCATGGCTACCACCGCCA	66.6	5	5	100.00%	0.729
SCoT30	CCATGGCTACCACCGGCG	64.2	10	10	100.00%	0.772
SCoT31	CCATGGCTACCACCGCCT	64.5	5	5	100.00%	0.743
SCoT34	ACCATGGCTACCACCGCA	65	4	4	100.00%	0.478
SCoT35	CATGGCTACCACCGGCCC	63.1	12	12	100.00%	0.825
SCoT36	GCAACAATGGCTACCACC	60	7	7	100.00%	0.793
Mean			6.957	6.913	99.46%	0.737

Note: OAT^1^, optimal annealing temperature; PIC^2^, polymorphism information content.

**Table 2:** Association analysis results between SCoT markers and phenotypic traits based on the MLM (*p* < 0.01).

Trait	Primer	Band	*p*-Value	*R* ^2^
Flowering duration	SCoT28	band1	0.0005	20.92%
Plant height	SCoT3	band3	0.0011	18.53%
Plant height	SCoT30	band8	0.0031	14.89%
Flowering duration	SCoT31	band1	0.0034	14.58%
Aphid resistance	SCoT35	band10	0.0052	13.32%
Peduncle diameter	SCoT20	band1	0.0069	12.31%
Flower color	SCoT14	band10	0.0079	11.81%
Leaf-miner resistance	SCoT36	band1	0.0082	11.18%

## Data Availability

The original contributions presented in the study are included in the article/Supplementary Materials. Further inquiries can be directed to the corresponding authors.

## Supplementary Materials

The following supporting information can be downloaded at: https://www.mdpi.com/article/10.3390/genes16060664/s1. Figure S1: Evaluation of *K*-values (*K* = 1–10) based on genetic structure analysis. Table S1: Phenotypic trait data for 65 chrysanthemum accessions. Table S2: Measurement protocols for trait characterization. Table S3: Variation analysis of seven phenotypic quantitative traits. Table S4: Pearson correlation coefficient matrix of 12 phenotypic traits. Table S5: Loading factors of 12 phenotypic traits based on PCA analysis.

## References

[1] Zhang S.L., Dai S.L. (2013). The Chinese Chrysanthemum Book.

[2] Su J., Jiang J., Zhang F., Liu Y., Ding L., Chen S., Chen F. (2019). Current achievements and future prospects in the genetic breeding of chrysanthemum: A review. Hortic. Res..

[3] Teixeira Da Silva J.A., Shinoyama H., Aida R., Matsushita Y., Raj S.K., Chen F. (2013). Chrysanthemum biotechnology: Quo vadis?. Crit. Rev. Plant Sci..

[4] Pan B., Pan B., Du Y., Chen Q., Chen Q., Wang Y., Chen L., Chen L., Li H., Huang C. (2025). China’s chrysanthemum in the global market: Evaluating the international competitiveness and influencing factors. Front. Sustain. Food Syst..

[5] Zhang X., Su J., Jia F., He Y., Liao Y., Wang Z., Jiang J., Guan Z., Fang W., Chen F. (2024). Genetic architecture and genomic prediction of plant height-related traits in chrysanthemum. Hortic. Res..

[6] Sun J., Wen C. (2025). Phenotypic variation and inheritance of leaf weight in cut chrysanthemum: Phenotypic variation and inheritance of leaf weight in cut chrysanthemum. Euphytica.

[7] Miler N., Jedrzejczyk I. (2018). Chrysanthemum plants regenerated from ovaries: A study on genetic and phenotypic variation. Turk. J. Bot..

[8] Lu C., Li Y., Wang J., Qu J., Chen Y., Chen X., Huang H., Dai S. (2021). Flower color classification and correlation between color space values with pigments in potted multiflora chrysanthemum. Sci. Hortic..

[9] Collard B.C.Y., Mackill D.J. (2009). Start codon targeted (SCoT) polymorphism: A simple, novel dna marker technique for generating gene-targeted markers in plants. Plant Mol. Biol. Rep..

[10] Al-Khayri J.M., Mahdy E., Taha H., Eldomiaty A.S., Abd-Elfattah M.A., Abdel L.A., Rezk A.A., Shehata W.F., Almaghasla M.I., Shalaby T.A. (2022). Genetic and morphological diversity assessment of five kalanchoe genotypes by SCoT, ISSR and RAPD-PCR markers. Plants.

[11] Deng L., Liang Q., He X., Luo C., Chen H., Qin Z. (2015). Investigation and analysis of genetic diversity of diospyros germplasms using SCoT molecular markers in guangxi. PLoS ONE.

[12] Rai M.K. (2023). Start codon targeted (SCoT) polymorphism marker in plant genome analysis: Current status and prospects. Planta.

[13] Shaban A.S., Arab S.A., Basuoni M.M., Abozahra M.S., Abdelkawy A.M., Mohamed M.M., Merah O. (2022). SCoT, ISSR, and SDS-PAGE investigation of genetic diversity in several egyptian wheat genotypes under normal and drought conditions. Int. J. Agron..

[14] Poczai P., Varga I., Laos M., Cseh A., Bell N., Valkonen J.P., Hyvönen J. (2013). Advances in plant gene-targeted and functional markers: A review. Plant Methods.

[15] Sumitomo K., Shirasawa K., Isobe S., Hirakawa H., Hisamatsu T., Nakano Y., Yagi M., Ohmiya A. (2019). Genome-wide association study overcomes the genome complexity in autohexaploid chrysanthemum and tags snp markers onto the flower color genes. Sci. Rep..

[16] Nguyen T.K., Ha S.T.T., Lim J.H. (2020). Analysis of chrysanthemum genetic diversity by genotyping-by-sequencing. Hortic. Environ. Biotechnol..

[17] Song X., Xu Y., Gao K., Fan G., Zhang F., Deng C., Dai S., Huang H., Xin H., Li Y. (2020). High-density genetic map construction and identification of loci controlling flower-type traits in chrysanthemum (*Chrysanthemum* × *morifolium* Ramat.). Hortic. Res..

[18] Li P., Zhang F., Chen S., Jiang J., Wang H., Su J., Fang W., Guan Z., Chen F. (2016). Genetic diversity, population structure and association analysis in cut chrysanthemum (*Chrysanthemum morifolium* Ramat.). Mol. Genet. Genom..

[19] Feng S., He R., Jiang M., Lu J., Shen X., Liu J., Wang Z., Wang H. (2016). Genetic diversity and relationships of medicinal chrysanthemum morifolium revealed by start codon targeted (SCoT) markers. Sci. Hortic..

[20] Samarina L.S., Malyarovskaya V.I., Reim S., Yakushina L.G., Koninskaya N.G., Klemeshova K.V., Shkhalakhova R.M., Matskiv A.O., Shurkina E.S., Gabueva T.Y. (2021). Transferability of ISSR, SCoT and SSR markers for *Chrysanthemum* × *morifolium* ramat and genetic relationships among commercial russian cultivars. Plants.

[21] Kulus D., Tymoszuk A., Gościnna K., Osial M. (2025). Enhancing germination and growth of chrysanthemum synthetic seeds through iron oxide nanoparticles and indole-3-acetic acid: Impact of treatment duration on metabolic activity and genetic stability. Nanotechnol. Sci. Appl..

[22] Tymoszuk A., Szałaj U., Wojnarowicz J., Kowalska J., Antkowiak M., Kulus D. (2024). Zinc oxide and silver effects on the growth, pigment content and genetic stability of chrysanthemums propagated by the node culture method. Folia Hortic..

[23] (2012). Guidelines for the Conduct of Tests for Distinctness, Uniformity, and Stability—Chrysanthemum.

[24] R Core Team (2023). R: A Language and Environment for Statistical Computing. Version 4.3.1, R Foundation for Statistical Computing.

[25] Nagy S., Poczai P., Cernák I., Gorji A.M., Hegedűs G., Taller J. (2012). PICcalc: An online program to calculate polymorphic information content for molecular genetic studies. Biochem. Genet..

[26] Liu K., Muse S.V. (2005). Powermarker: An integrated analysis environment for genetic marker analysis. Bioinformatics.

[27] Kumar S., Stecher G., Tamura K. (2016). MEGA7: Molecular evolutionary genetics analysis version 7.0 for bigger datasets. Mol. Biol. Evol..

[28] Falush D., Stephens M., Pritchard J.K. (2007). Inference of population structure using multilocus genotype data: Dominant markers and null alleles. Mol. Ecol. Notes.

[29] Earl D.A., Vonholdt B.M. (2012). Structure harvester: A website and program for visualizing structure output and implementing the evanno method. Conserv. Genet. Resour..

[30] Jakobsson M., Rosenberg N.A. (2007). CLUMPP: A cluster matching and permutation program for dealing with label switching and multimodality in analysis of population structure. Bioinformatics.

[31] Peakall R., Smouse P.E. (2012). Genalex 6.5: Genetic analysis in excel. Population genetic software for teaching and research—An update. Bioinformatics.

[32] Bradbury P.J., Zhang Z., Kroon D.E., Casstevens T.M., Ramdoss Y., Buckler E.S. (2007). Tassel: Software for association mapping of complex traits in diverse samples. Bioinformatics.

[33] Hodaei M., Rahimmalek M., Arzani A. (2019). Genetic diversity of iranian *Chrysanthemum morifolium* cultivars using morphological traits and sequence-related amplified polymorphism (SRAP) markers. Hortic. Environ. Biotechnol..

[34] Roein Z., Hassanpour Asil M., Sabouri A., Dadras A.R. (2014). Genetic structure of chrysanthemum genotypes from iran assessed by AFLP markers and phenotypic traits. Plant Syst. Evol..

[35] Fu X., Su J., Yu K., Cai Y., Zhang F., Chen S., Fang W., Fadi C., Guan Z. (2018). Genetic variation and association mapping of aphid (*Macrosiphoniella sanbourni*) resistance in chrysanthemum (*Chrysanthemum morifolium* ramat.). Euphytica.

[36] Yang X., Su J., Qu Y., Jiang J., Guan Z., Fang W., Chen F., Zhang F. (2023). Dissecting the inheritance pattern of the anemone flower type and tubular floral traits of chrysanthemum in segregating f1 populations. Euphytica.

[37] Zhang F., Chen S., Chen F., Fang W., Deng Y., Chang Q., Liu P. (2011). Genetic analysis and associated SRAP markers for flowering traits of chrysanthemum (*Chrysanthemum morifolium*). Euphytica.

[38] Zhou Y., Wei X., Abbas F., Yu Y., Yu R., Fan Y. (2021). Genome-wide identification of simple sequence repeats and assessment of genetic diversity in *Hedychium*. J. Appl. Res. Med. Aromat. Plants.

[39] Su J., Zhang F., Li P., Guan Z., Fang W., Chen F. (2016). Genetic variation and association mapping of waterlogging tolerance in chrysanthemum. Planta.

[40] Su J., Zhang F., Chong X., Song A., Guan Z., Fang W., Chen F. (2019). Genome-wide association study identifies favorable snp alleles and candidate genes for waterlogging tolerance in chrysanthemums. Hortic. Res..

[41] Li P., Su J., Guan Z., Fang W., Chen F., Zhang F. (2018). Association analysis of drought tolerance in cut chrysanthemum (*Chrysanthemum morifolium* Ramat.) At seedling stage. 3 Biotech.

[42] Wan W., Jia F., Liu Z., Sun W., Zhang X., Su J., Guan Z., Chen F., Zhang F., Fang W. (2024). Quantitative evaluation and genome-wide association studies of chrysanthemum flower color. Sci. Hortic.

[43] Su J., Zeng J., Wang S., Zhang X., Zhao L., Wen S., Zhang F., Jiang J., Chen F. (2024). Multi-locus genome-wide association studies reveal the dynamic genetic architecture of flowering time in chrysanthemum. Plant Cell Rep..

[44] Zhang X., Ning X., He Y., Su J., Wen S., Lu Z., Sun W., Wang H., Guan Z., Fang W. (2025). GWAS reveals the genetic basis and genomic regions underlying four active compounds in chrysanthemum. Hortic. Plant J..

