# Supplementary figures and images for: Phenotypic Characterization and Marker–Trait Association Analysis Using SCoT Markers in Chrysanthemum (Chrysanthemum morifolium Ramat.) Germplasm
Source: Genes (Basel). 2025 May 29;16(6):664. doi: 10.3390/genes16060664 (PMC12192018; doi:10.3390/genes16060664)

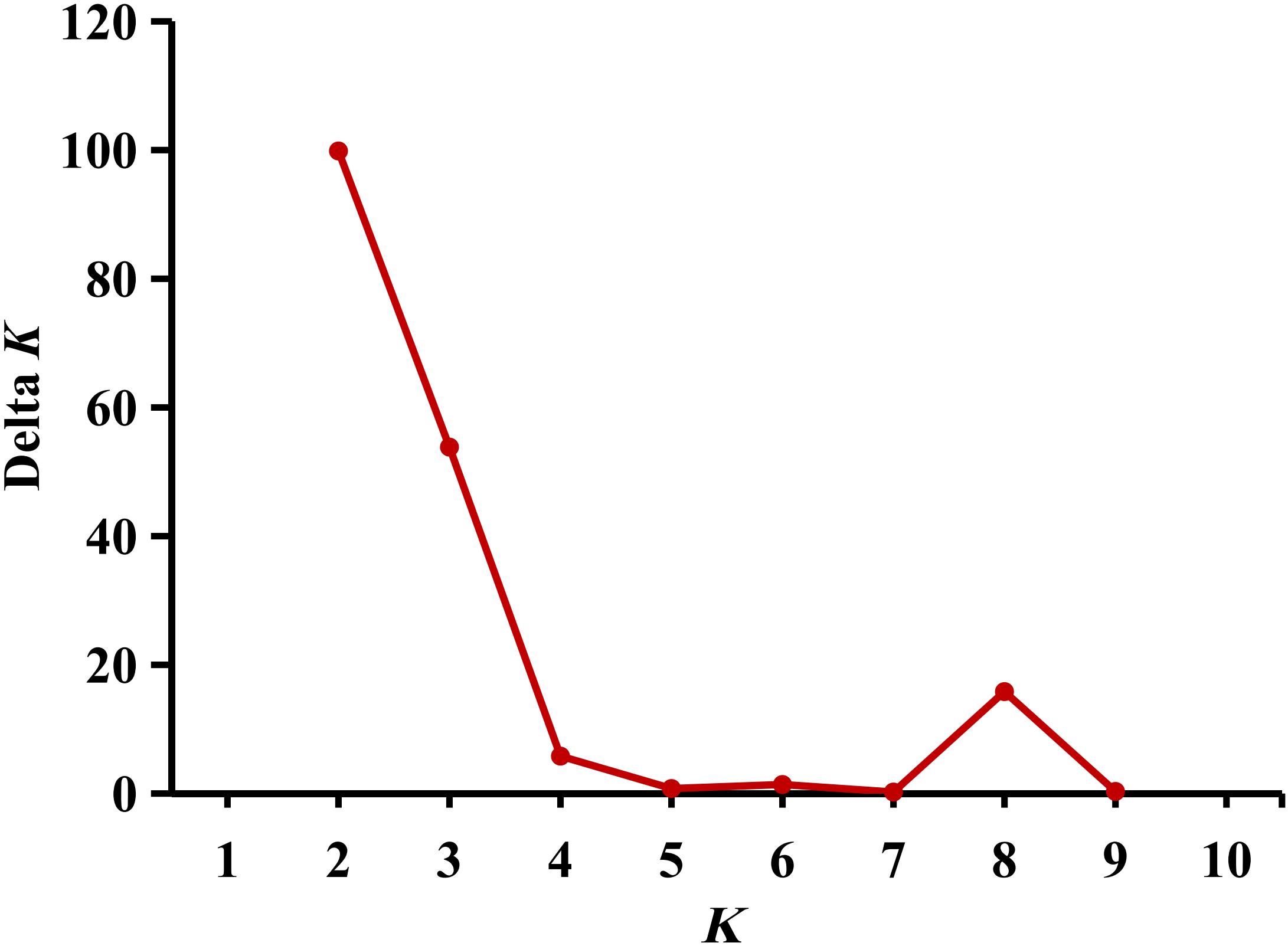

Supplement: Supplementary file 1 [file genes-16-00664-s001.zip › Figure S1.jpg]
